# Protein Labelling Accuracy for UK Patients with PKU Following a Low Protein Diet

**DOI:** 10.3390/nu12113440

**Published:** 2020-11-10

**Authors:** Dilyana Kraleva, Sharon Evans, Alex Pinto, Anne Daly, Catherine Ashmore, Kiri Pointon-Bell, Júlio César Rocha, Anita MacDonald

**Affiliations:** 1 Faculty of Health, Education & Life Sciences, Birmingham City University City South Campus, Westbourne Road, Edgbaston, Birmingham B15 3TN, UK; didincee@gmail.com (D.K.); Kiri.Pointon-Bell@bcu.ac.uk (K.P.-B.); 2 Birmingham Women’s and Children’s NHS Foundation Trust, Steelhouse Lane, Birmingham B4 6NH, UK; Sharon.morris6@nhs.net (S.E.); alex.pinto@nhs.net (A.P.); a.daly3@nhs.net (A.D.); Catherine.ashmore@nhs.net (C.A.); 3 Nutrition & Metabolism, NOVA Medical School, Faculdade de Ciências Médicas, Universidade Nova de Lisboa, Campo Mártires da Pátria, 130, 1169-056 Lisbon, Portugal; rochajc@nms.unl.pt; 4 Center for Health Technology and Services Research (CINTESIS), R. Dr. Plácido da Costa, s/n, 4200-450 Porto, Portugal

**Keywords:** phenylketonuria, food labelling, protein content, free from, gluten free

## Abstract

A phenylalanine (protein)-restricted diet is the primary treatment for phenylketonuria (PKU). Patients are dependent on food protein labelling to successfully manage their condition. We evaluated the accuracy of protein labelling on packaged manufactured foods from supermarket websites for foods that may be eaten as part of a phenylalanine-restricted diet. Protein labelling information was evaluated for 462 food items (“free from”, *n* = 159, regular, *n* = 303), divided into 16 food groups using supermarket website data. Data collection included protein content per portion/100 g when food was “as sold”, “cooked” or “prepared”; cooking methods, and preparation instructions. Labelling errors affecting protein content were observed in every food group, with overall protein labelling unclear in 55% (*n* = 255/462) of foods. There was misleading, omitted, or erroneous (MOE) information in 43% (*n* = 68/159) of “free from” foods compared with 62% (*n* = 187/303) of regular foods, with fewer inaccuracies in “free from” food labelling (*p* = 0.007). Protein analysis was available for uncooked weight only but not cooked weight for 58% (*n* = 85/146) of foods; 4% (*n* = 17/462) had misleading protein content. There was a high rate of incomplete, misleading, or inaccurate data affecting the interpretation of the protein content of food items on supermarket websites. This could adversely affect metabolic control of patients with PKU and warrants serious consideration.

## 1. Introduction

Phenylketonuria (PKU) is a rare, autosomal recessive inborn error of metabolism due to low or absent activity of the enzyme phenylalanine hydroxylase (PAH), required for degradation of phenylalanine to tyrosine. It causes elevated levels of phenylalanine in the blood and brain and if untreated, leads to severe, irreversible, intellectual disability [1]. Maintaining low blood phenylalanine levels within defined target ranges prevents phenylalanine toxicity [1]. Although it can be managed with a combined approach of dietary and pharmaceutical treatment, the only treatment option in the UK is a lifelong, phenylalanine-restricted diet [2]. Dietary management is stringent, requiring discipline and tenacity, and it is well established that many patients with PKU of all ages are unable to sustain satisfactory blood phenylalanine control [3,4]. Although there are multiple causes for unsatisfactory metabolic control, relatively small deviations from dietary prescription can adversely affect blood phenylalanine levels in patients with classical PKU [5].

Dietary treatment involves the avoidance of high-protein foods such as meat, fish, eggs, cheese, seeds, soya, and nuts and a limited intake of natural protein from foods such as cereals, potatoes, milk, and some vegetables. Any natural protein intake should be calculated, measured, and controlled and up to 80% of patients tolerate <10 g/day [3]. Fruit and vegetables with a phenylalanine content ≤75 mg/100 g, butter, oils, and sugars are given without restriction [2]. Dietary protein is supplemented with synthetic protein, either phenylalanine-free amino acids or low-phenylalanine glycomacropeptide, with added vitamins and minerals to meet nutritional protein requirements. Caregivers and people with PKU are trained in reading and interpreting the protein amounts on manufactured food labels. They are reliant on supermarkets and manufacturers to provide accurate and easily interpreted information about protein content on food labels. Almost every UK supermarket offers an online food delivery service and survey data suggest that 29% of people purchase food via online shopping [6], with online grocery shopping available in at least 60 countries worldwide [7]. Website supermarket shopping is popular for those with special dietary requirements, giving the opportunity to examine food labels prior to food purchase [8]. Patients with PKU and their families can browse the protein nutritional analysis of foods and examine information about food preparation, cooking, and reconstitution of foods. Some online supermarket websites are intuitive to dietary needs and can even create a specific dietary profile that will highlight products that should be avoided for food allergies, although the needs of patients with PKU are not considered [9].

On 25 October 2011, the European Parliament and Council adopted Regulation (EU) No 1169/2011 that issued legal standards for the labelling and information given to consumers by food manufacturers (called the “Food Information to Consumers (FIC) Regulation”) [10]. This regulation has been applied since 2016. When pre-packaged foods are sold “online”, it is regulated that the responsibility for providing mandatory food information (except the date of minimum durability or the “use by” date) sits with the owner of the online website (the responsible food business operator). Online pre-packaged mandatory food information should include information on the weight and volume of food (net quantity information), a list of ingredients, protein content per 100 g/100 mL, and instructions for use or cooking, if applicable. For non-packaged food, there are fewer rigid stipulations, but the food business operator is required to provide allergen information [11].

In PKU, if foods are eaten because of inaccurate or ambiguous website or food labelling information it may cause unexplained, poor blood phenylalanine control. In countries like the UK, there is high reliance on manufactured foods, so reliability of food labelling information is particularly important. Inadequate, misleading, or unclear information about protein content may deter caregivers/patients with PKU from purchasing specific foods. Therefore, it is in the best interests of manufacturers to supply suitable and trustworthy information that is easy to understand and accurate. The clarity of food labelling and food information on online supermarket websites remains unstudied for people with special dietary requirements such as PKU.

The aim of this study was to evaluate the accuracy of protein labelling on packaged manufactured foods from supermarket websites for foods that may be eaten as part of a phenylalanine-restricted diet. Patients with PKU may use some “free from foods”, particularly gluten free, which may have a lower protein content than foods containing wheat or milk, so emphasis was placed on this group of foods.

## 2. Materials and Methods

From January 2019 to April 2020, 462 packaged food items were examined using descriptive information given by major UK supermarkets (Asda, Morrisons, Sainsbury’s, Tesco, and Waitrose) available on their website. For each food, factors that may alter or affect the protein analysis were tabulated. The selection of foods was not random as this was conducted based on food popularity and common usage. Foods were chosen based on their potential suitability in a protein-restricted diet and mainly had a protein content <10 g/100 g. A selection of “free from” (all gluten-free) and regular foods were examined. Both commercial branded products and the supermarkets’ own brands were included. Meat and fish products were avoided, although two types of regular cheese were included to compare information with “free from” varieties. Items were divided and analysed by the following food groups: bread and bread products, breakfast cereals, vegan cheese, cakes, sweet biscuits, pastries/tarts, crackers, chocolate, crisps, desserts, flours, gravies/sauces, pasta, vegetable foods, dried pot noodles and yoghurts. The supermarket websites accessed were required to give a product description and nutritional analysis for each food.

The following data were collected for each food item: product description, ingredients, preparation, cooking instructions and usage, net and portion size. Specific information collected about protein content included: protein content per portion as cooked/prepared, protein content per portion as sold; protein content per 100 g expressed as cooked/prepared, protein content per 100 g as sold; any misleading information about protein content per 100 g or per food portion (e.g., when a food protein content states 0.0 g per portion, but was >0.1 g/100 g or if the protein content for each portion was only described as <0.5 g with no other relevant information, or if there was a discrepancy between the ingredients listed and the protein content); reconstitution instructions for dry powders, including protein content per portion/100 g supplied when protein analysis was given after dry products had been reconstituted.

All information was transferred onto a database and coded according to the accuracy of information. All products were checked twice by two different dietitians for accuracy and to minimise any risk of bias. Statistical analysis was performed using Mann–Whitney unpaired *t*-tests to compare numbers of misleading, omitted, or erroneous (MOE) foods in “free from” and “regular” food groups and to compare types of MOE information between the two groups. Percentage error in “free from” and “regular” foods were also compared using Wilcoxon signed-rank tests.

## 3. Results

### 3.1. Accuracy of Product Description

The product description, ingredients, net weight, portion size, protein content per 100 g cooked and uncooked, and preparation and cooking instructions were checked for 462 foods from five supermarket websites (Asda, Morrisons, Sainsbury’s, Tesco, and Waitrose). There were 159 “free from” foods and 303 “regular” foods (Table 1). All the “free from” foods were gluten free. Overall, 255 of 462 (55%) foods had information that was MOE from the website product information given by supermarkets, thereby affecting the interpretation of protein content for food items. There were fewer inaccuracies in “free from foods” (MOE, 68 of *n* = 159 foods, 43%), compared with regular foods (MOE, 187 of *n* = 303 foods, 62%) (*p* = 0.007, Wilcoxon signed-rank test) most notably for breads, bread products, and flours.

### 3.2. Types of Misleading Omitted, or Erroneous (MOE) Information in Food Product Information that Affected Protein Content

All types of MOE information are categorised by issue in Table 2. Some food items had more than one descriptive issue that affected interpretation of their protein content.

#### 3.2.1. Food Label did not Distinguish if Protein Content was for Food Item when Cooked/Prepared or as Sold

Thirty two percent (*n* =146/462) of foods required further cooking or preparation. Of these 7% (*n* = 10/146) did not specify whether the protein analysis per 100 g/weight of food was for the cooked/prepared or ‘as sold’ product. When expressed per portion size this figure was 14% (*n* = 20/146).

#### 3.2.2. Missing Information about Protein Content per 100 g (either Omitted Protein Content for Cooked/Prepared Weight or for Uncooked/Unprepared Weight)

Protein content was given for uncooked weight only but not cooked weight for 58% (*n* = 85/146) of foods requiring preparation. In contrast, 33% (*n* = 48/146) of foods gave protein value for cooked but not uncooked weight. These issues were more commonly “regular” foods than “free from” foods (*p* = 0.03 and 0.02 respectively).

#### 3.2.3. Missing Information about Protein Content per Portion Size

Twenty-seven percent (*n* = 125/462) of foods did not give a weight for an estimated portion/serving size; these were more likely to be “regular” foods than “free from” foods (*p* = 0.001). Ten percent (*n* = 47/462) of foods did not give the protein analysis of the portion size. Protein content was omitted per cooked portion size in 35% (*n* = 51/146) of foods or omitted per portion as sold in 26% (*n* = 38/146).

#### 3.2.4. Omitted Cooking Instructions

This information was omitted in 8% (*n* = 12/146) of food items.

#### 3.2.5. Missing Net Size

This information was omitted in 5% of foods (*n* = 21/462).

#### 3.2.6. Misleading/Incorrect Protein Content

Four percent (*n* = 17/462) of foods had misleading protein analysis. Either the protein content per portion size stated 0 g protein when the analysis per 100 g stated a protein content >0.0 g (more commonly in “regular” foods than “free from” foods; *p* = 0.004), or the protein content per 100 g or per portion stated <0.5 g but did not give a specific protein amount. One food had an incorrect protein analysis; this product contained 70% peas, and it was stated that it contained a protein content of only 0.5 g/100 g when it should have contained around 4 g/100 g.

#### 3.2.7. Missing Protein Analysis

Two foods contained no protein analysis (a jelly and frozen potato product). These products were produced by the same manufacturer.

#### 3.2.8. Preparation/Reconstitution Information

Twelve percent (*n* = 18/146) of foods requiring preparation gave the protein analysis only after a product had been reconstituted/prepared with milk even though milk was not part of the ingredients list. Consequently, this “theoretical” protein analysis portrayed these foods to be unsuitable in a low-protein diet. The protein content of the dry ingredients was not given. This was more likely to occur in “regular” foods than in “free from” foods although it did not reach statistical significance.

#### 3.2.9. Omitted Ingredients List

Two percent (*n* = 9/462) of foods did not give an ingredients list.

### 3.3. Frequency of Misleading, Omitted, or Erroneous (MOE) Food Information for Food Groups and for Individual Foods

Thirty eight percent (*n* = 96/255) of foods with MOE information had one inaccuracy, 37% (*n* = 95/255) had two inaccuracies, and 16% (*n* = 41/255) had three inaccuracies regarding their information, which affected the interpretation of the food protein content. Bread and bread products, cake, and biscuits commonly had missing information about portion sizes. Pasta and vegetable products regularly had omitted information about the protein content for cooked or uncooked product (either per 100 g or per portion size). Pot noodles were particularly misleading; their protein content was commonly given per 100 g reconstituted weight rather than dry weight, but this was unclear. The protein content of “regular” custards, instant desserts, and some “regular” and “free from” cereals were only given after reconstitution with milk, and commonly had unclear portion sizes.

**Figure 1:**
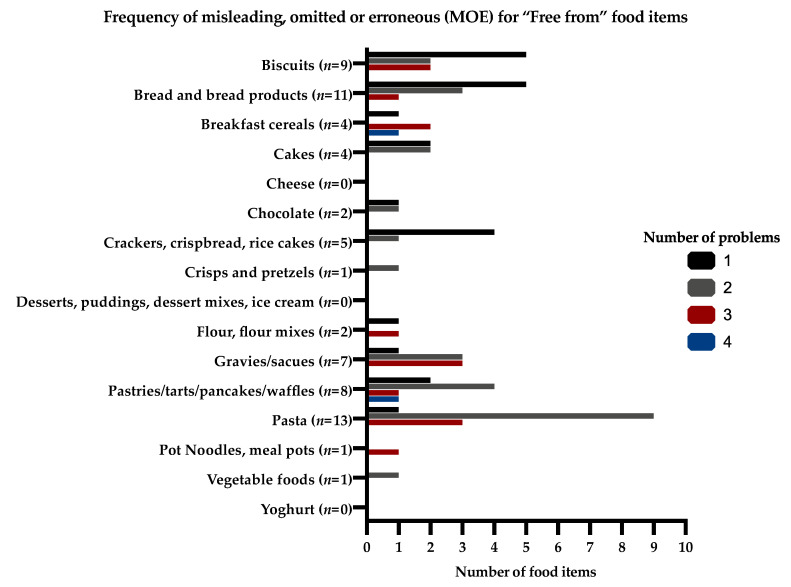
Frequency of misleading, omitted, or erroneous (MOE) information for “free from” food items that would affect their protein content given on the supermarket websites.

**Figure 2:**
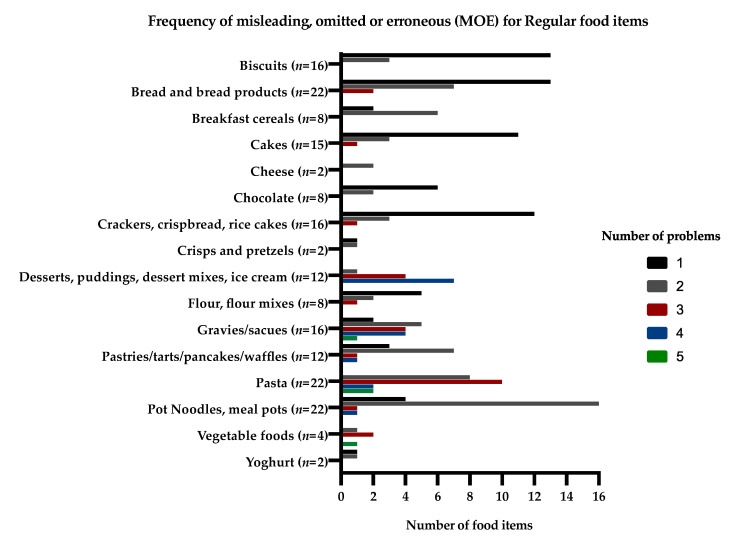
Frequency of misleading, omitted, or erroneous (MOE) information for regular food items that would affect their protein content given on the supermarket websites.

The frequency of MOE information and the number of problems for the same food items (“free from” and “regular” food items) that would affect their protein content given on the supermarket websites are presented in Figure 1 and Figure 2.

## 4. Discussion

This research indicates that interpreting the protein content for some common supermarket foods available via online websites is inadequate, unclear and even misleading for people with PKU. Information about the protein content per portion size was sometimes omitted or indeterminate, particularly for “regular” foods compared with “free from” foods. For “regular” dried products requiring reconstitution, the protein content was commonly given only after the product has been prepared with “added” cow’s milk, which then increased the protein content of the food, rendering it unsuitable for most people with PKU. For other products consisting of dry ingredients, it was sometimes uncertain if protein labelling was for the dry product or after preparation. For products such as gluten-free biscuits, the protein content was stated as <0.5 g per portion only, even though the protein per 100 g was much higher, and the food item included protein-containing ingredients. Not all products identified net weight.

Food regulations, manufacturers, and online food business operators have not considered the impact of any inaccurate product information for people on very low-protein diets. Fortunately, mandatory FIC nutrition labelling for pre-packaged foods does include protein content, but it is listed only after energy, fat (including saturates), and carbohydrates (including sugar). For non–prepacked foods, there is no requirement in the EU FIC regulations for any nutrition information to be provided, but many manufacturers voluntarily declare the protein content. The FIC regulations states that food manufacturers are not required to do their own laboratory analysis for protein content and it is possible for a food business operator to calculate the values themselves (1) from the known or actual average values of the ingredients used or (2) from generally established and accepted data [12]. The accuracy of protein measurement by these methods is unknown and the definition of what is meant by “generally established and accepted” data is not given, so manufacturers could interpret this in different ways. It is also unknown how many food businesses estimate the protein content by using published protein values of similar foods rather than estimating individual foods by chemical analysis.

Some patients with PKU tolerate a minimal amount of protein (3 to 4 g/day) so accurate protein information is crucial [13,14,15]. In conflict, the FIC regulations apply protein tolerances to food labels on the basis that protein analysis is not precise due to natural variations in ingredient composition and changes in production. They appear unaware of the needs of patients on very low-protein diets. For foods containing protein <10 g/100 g, they state that the protein content may be within ±2 g; for foods containing protein 10–40 g/100 g, the protein content is ±20%; and for foods containing protein >40 g per 100 g, protein content is within ±8 g [16,17]. Additionally, rounding guidance suggested by the EU states that for food containing protein ≥10 g/100 g or 100 mL, the protein should be declared to the nearest 1 g (no decimals); protein between <10 g and >0.5 g/100 g or mL to the nearest 0.1 g; and protein at ≤0.5 g/100 g or mL as “0 g” or “<0.5 g.” We identified eight foods, particularly “free from” items, that stated that the food portion contained <0.5 g protein, even though each portion could have contained 0.4 g protein (<0.5 g); this amount would need to be calculated in a very low-protein diet as it may impact on metabolic control. Some patients with PKU have unexplained fluctuating daily blood phenylalanine levels and some of this may be due to the approximate nature of food protein labelling [18].

The FIC regulations state that the nutrition declaration is required for the food as sold, but, instead and where appropriate, it can relate to the food as prepared, provided sufficiently detailed preparation instructions are given. It is therefore possible to include only the nutrition information “as prepared” for foods such as dehydrated powdered soup or desserts. This is deceptive and unsafe for people with PKU. Commonly, we found that nutrition labels for “regular” dessert mixes were calculated based on their preparation with cow’s milk, and this should be avoided in PKU. The addition of milk substantially increased the protein content, even though many of the raw ingredients of dessert mixes were low in protein. By declaring protein content after preparation with other added ingredients, products appear unsuitable for patients on a low-protein diet, even though it may have been possible to consume the food product if it had been made up with a low-protein milk alternative. Additionally, giving the protein content of pot noodles after preparation is confusing and this has led to several incidents when caregivers/patients have miscalculated and underestimated their protein content [19].

It was common for supermarket websites to omit information about whether the protein analysis was associated with cooked or uncooked food. The protein content of a food product will vary depending on if it is dry cooked, fried, microwaved, or uncooked [20]. Commonly, foods such as potatoes have a high water content, and dry cooking results in moisture loss and a more concentrated protein amount [20]. These protein differences must be considered in a low-protein diet. The FIC regulations state that instructions on how to prepare and cook the food, including heating in a microwave oven, must be given on the label if they are needed [21,22,23]. If the food must be heated, the temperature of the oven and the cooking time should usually be stated, so it is sensible to give food analysis both for the “as sold” state and for “cooked”, as recommended.

The protein content of a portion size was either omitted or the portion size was not quantified by weight for 172 of 462 foods (37%), particularly in “regular” foods, contributing to the difficulty in calculating the protein content of foods consumed in a phenylalanine-restricted diet. The FIC regulations state that the portion or consumption unit should be easily recognisable by the consumer, quantified on the label in close proximity to the nutrition declaration, and the number of portions or units contained in the package must be stated on the label. The “consumption unit” information requires improvement.

Legislation on food labelling gives instruction to producers and retailers; it also gives the consumers rights to basic information. We have shown that the information on the protein content of foods via supermarket websites is inaccurate and potentially harmful to those with PKU. Unlike allergies, there is little understanding of the essential role of a very low-protein diet and the harmful impact of poor control on patients’ neuropsychological health [9]. This may also apply to other patients with inherited disorders of protein metabolism such as Maple Syrup Urine Disease or Tyrosinaemia type I or II. They also rely on accurate food labelling to manage their dietary treatment safely.

There appears to be no audit or regular assessment of supermarket websites to check accuracy of information that is provided to the consumer. Manufacturers should indicate on food labels how they have estimated protein content. We identified a packaged food product containing 70% peas and 30% carrots (with no other added ingredients), but it stated that it only contained protein 0.5 g/100 g, when it should have contained a protein amount of around 4 g/100 g (based on the established protein content of peas). For children to be given a food they enjoy in error, leads to additional psychological stress and guilt for the parents. We identified two other packaged products made by the same company without any protein analysis. These products were targeted at young children, so likely to be mistakenly eaten by a population very vulnerable to the impact of high blood phenylalanine concentrations.

This study did have some limitations. Although almost 500 foods were examined, matched numbers and types of “free from” foods were not compared with regular foods. However, as an overall group, “free from” foods website supermarket information gave more comprehensive data that would enable the consumer to assess the protein content of the product consumed. This was commonly due to the low availability of some “free from” foods, such as pot noodles or dessert pots. There was not an equal number of foods examined in all the different food groups, with small numbers of the following products examined: cheese, yoghurts, ice cream, and vegetable products. Food products in this study were not chosen by random, but commonly selected in order of popularity and usage, so it is accepted that there are limitations in product selection, especially with gluten-free or “free from” products that are usually purchased by patients with coeliac disease or food intolerance. Website information was not compared with product labelling on packages as purchased from the supermarket shelves, which may have identified further discrepancies.

## 5. Conclusions

Obtaining accurate information about the protein content of some foods from online supermarket website information is challenging. A high proportion of incomplete, misleading, or inaccurate data was identified that directly affected the interpretation of the protein content of food items. Inadequate protein food labelling is likely to contribute to the difficulties in maintaining good metabolic control in PKU. It is important that all dietitians, patients, and families of patients with PKU are aware of the food label limitations and potential problems.

Although food producers and business operators are expected to provide information to consumers that is clear and accurate, little attention is paid to the exactness of protein food labelling. The FIC regulations should be reconsidered, with more attention given to monitoring the accuracy of information provided by supermarket websites. Poor awareness of the impact and inattention to the factors that affect food protein content and carelessness about the accuracy of protein labelling can adversely affect the neurological health of people with PKU and deserves urgent consideration.

## Figures and Tables

**Table 1:** Number of individual foods with misleading, omitted, or erroneous (MOE) information that would affect the protein content calculation from information given on the supermarket websites.

	“Free From/Gluten-Free” Foods (*n* = 159)		“Regular” Foods (*n* = 303)		*p* Value *
Food Groups	Number of Foods Examined	Number of Foods with MOE Issues (%)	Number of Foods Examined	Number of Foods with MOE Issues (%)	
Biscuits	29	9 (31)	48	16 (33)	>0.99
Bread and bread products	33	11 (33)	35	22 (63)	0.02
Breakfast cereals	7	4 (57)	13	8 (62)	>0.99
Cakes	14	4 (28)	27	15 (56)	0.19
Cheese	1	0 (0)	2	2 (100)	ID
Chocolate	13	2 (15)	26	8 (31)	0.45
Crackers, crispbread, rice cakes	17	5 (29)	34	16 (47)	0.37
Crisps, pretzels	2	1 (50)	4	2 (50)	>0.99
Desserts, puddings, dessert mixes, ice cream	2	0 (0)	20	12 (60)	0.19
Flours, flour mixes	4	2 (50)	8	8 (100)	0.09
Gravies/sauces	8	7 (88)	16	16 (100)	0.33
Pastries/tarts/pancakes/waffles	11	8 (73)	16	12 (75)	>0.99
Pasta	13	13 (100)	22	22 (100)	>0.99
^$^ Pot noodles, meal pots	1	1 (100)	22	22 (100)	ID
Vegetable foods	1	1 (100)	4	4 (100)	ID
Yogurt	3	0 (0)	6	2 (33)	0.50
Total food numbers	159	68 (43)	303	187 (62)	

Abbreviations: MOE, foods with misleading, omitted, or erroneous information. * Mann–Whitney unpaired *t*-test; ^$^ Pot noodles: a mix of dehydrated noodles, assorted dried vegetables and flavouring powder in a pot. They are prepared by adding boiling water. ID = insufficient data.

**Table 2:** Type of misleading, omitted, or erroneous (MOE) information that would affect protein content describing individual foods given on the supermarket websites.

	All Foods	“Free From” Foods	“Regular” Foods	*p* Value *
Issue	*n* = 462 (%)	*n* = 159 (%)	*n* = 303 (%)	
Unspecified if protein content given per food portion is for cooked/prepared or weight as sold	20 (14) **	9 (22) **	11 (10) **	0.11
Unspecified if protein content given per 100 g is for cooked/prepared or weight as sold	10 (7) **	3 (2) **	7 (7) **	>0.99
Protein amount given is the same per 100 g and per portion (but one portion does not weigh 100 g)	1 (<1)	0 (0)	1 (<1)	0.47
Cooking/preparation instructions missing	12 (8) **	5 (12) **	7 (7) **	0.32
Protein content per 100 g cooked/prepared missing	85 (58) **	30 (73) **	55 (52) **	0.03
Protein content per 100 g uncooked/unprepared missing	48 (33) **	7 (17) **	41 (39) **	0.02
Protein content per portion size missing	47 (10)	16 (10)	31 (10)	0.96
Protein content per portion size cooked/prepared missing	51 (35) **	14 (34) **	37 (35) **	>0.99
Protein content per portion as sold missing but provided when cooked/prepared	38 (26) **	3 (7) **	35 (33) **	0.001
Weight of portion size missing	125 (27)	28 (18)	97 (32)	0.001
Missing net size	21 (5)	5 (3)	16 (5)	0.30
Protein content states 0 g per portion, even though contains protein > 0.1 g/100 g	7 (2)	6 (4)	1 (<1)	0.004
Protein content per portion described as <0.5 g	8 (2)	1 (1)	7 (2)	0.19
Protein content per 100 g described as <0.5 g protein	2 (<1)	0 (0)	2 (1)	0.31
Incorrect protein analysis	1 (<1)	0 (0)	1 (<1)	0.47
Missing protein analysis	2 (<1)	0 (0)	2 (1)	0.31
Protein content per portion only after prepared with milk	10 (7) **	0 (0)	10 (10) **	0.06
Protein content per 100 g only after prepared with milk	8 (5) **	0 (0)	8 (8) **	0.11
Missing ingredients list	9 (2)	1 (1)	8 (3)	0.14

* Mann–Whitney unpaired *t*-test. ** total number of foods requiring cooking/preparation *n* = 146; free from *n* = 41, regular *n* = 105.
